# Croatian freshwater bryoflora–diversity and distribution

**DOI:** 10.3897/BDJ.10.e83902

**Published:** 2022-05-25

**Authors:** Anja Rimac, Vedran Šegota, Antun Alegro, Nina Vuković, Nikola Koletić

**Affiliations:** 1 Department of Botany, Division of Biology, Faculty of Science, University of Zagreb, Zagreb, Croatia Department of Botany, Division of Biology, Faculty of Science, University of Zagreb Zagreb Croatia

**Keywords:** aquatic bryophytes, liverworts, mosses, freshwater habitats, rivers, lakes, chorology, southeast Europe

## Abstract

An extensive macrophyte field survey of running and standing waters was conducted from 2016 to 2021 at 786 sampling sites across Croatia as a part of the implementation of the Water Framework Directive. This survey is the first to present a comprehensive floristic catalogue of the freshwater bryoflora, along with an analysis of the distribution and diversity patterns on a national level. In all, 83 bryophyte species (68 mosses and 15 liverworts) were recorded in the 228 sites, with average species richness of 4.17 species per site. The most frequent species were *Fontinalisantipyretica*, *Rhynchostegiumriparioides*, *Leptodictyumriparium* and *Cratoneuronfilicinum*. The majority of the species encountered were rarely found, with over 70% of species recorded on less than 10 sampling sites and the majority of the species not being truly aquatic, rather being classified as facultative aquatics. The Dinaric Ecoregion, characterised by clean, cold, fast-flowing karstic rivers, especially in the Continental Subecoregion, supported higher freshwater bryophyte diversity than the lowland Pannonian Ecoregion, with mostly slow, eutrophic lowland watercourses with unstable sandy and gravelly alluvial sediments. Chorological comparison of Croatian eco- and subecoregions revealed the expected dominance of circumpolar and European elements, i.e. temperate chorotypes, as well as some biogeographical differences. The most frequent life forms were aquatic trailings and turfs. Amongst the recorded species, perennials and colonists were the most represented life strategies. The analysis of both the life-form and life-strategy spectra showed some differences amongst the Croatian regions, supporting the fact that the Dinaric Ecoregion provides more truly aquatic habitats and microhabitats suitable for the freshwater bryophytes, while in the Pannonian Ecoregion freshwater bryophytes dominantly inhabit the periodically submerged riparian zones, for example shaded lowland forest streams and rivulets or gently sloping margins of rivers and lakes.

## Introduction

Bryophytes colonised aquatic and riparian environments through several independent phylogenetical lineages of terrestrial species, by a secondary process of colonisation and morphological and physiological adaptations to a highly specialised habitat (Vitt and Glime 1984, Akiyama 1995, Cook 1999). These bryophytes inhabit various aquatic and riparian habitats, from mires, ponds and lakes to streams and rivers, as well as an ample range of hydrological niches associated with these habitat types along two major environmental gradients – water flow and water level fluctuations (Vitt and Glime 1984). However, they failed to conquer saltwater environments, with only a few species tolerating intertidal cycles and none living submerged (Vitt and Glime 1984). On a larger scale, the diversity and community structure of bryophytes associated with freshwater habitats is governed not only by hydrological and hydromorphological, but also by geological and climatological factors, as well as by water chemistry and land use of the catchment area (Suren 1996, Suren and Ormerod 1998, Scarlett and O'Hare 2006, Tremp et al. 2012, Gecheva et al. 2017, Vieira et al. 2018). The presence and cover of bryophytes in freshwater habitats are primarily determined by riverbed stability and substrate size (Suren 1996, Scarlett and O'Hare 2006, Tremp et al. 2012) with bryophytes being the dominant component of the macrophyte vegetation within watercourses that provide large and stable substrates, such as source areas, headwater and mountain streams, as well as waterfalls (Vitt et al. 1986, Zechmeister and Mucina 1994, Suren 1996, Tremp et al. 2012, Ceschin et al. 2015, Mucina et al. 2016). Here, the other macrophyte groups, especially vascular plants, are almost completely absent, primarily because of the fast and turbulent flow, rocky substrates, steep slopes and low temperatures. Furthermore, bryophytes thrive in highly seasonal and intermittent rivers due to their wide variety of adaptations enabling desiccation tolerance and ability to withstand dry periods. Therefore, bryophytes play a vital and sometimes dominant role in freshwater ecosystems, constituting a significant part of macrophyte communities, acting as primary producers, having profound influences on nearly all aspects of nutrient and organic matter processing in streams, providing food and shelter for macroinvertebrates, as well as an epiphytic habitat of rich periphyton communities (Stream Bryophyte Group 1999).

The studies on bryophytes of aquatic and semi-aquatic habitats so far conducted in Europe (e.g. Muotka and Virtanen 1995, Papp 1999, Vanderpoorten and Klein 1999b, Papp et al. 2006, Scarlett and O'Hare 2006, Gecheva et al. 2010, Ceschin et al. 2012a, Ceschin et al. 2012b, Vieira et al. 2014, Ceschin et al. 2015, Gecheva et al. 2017, Vieira et al. 2018), as well in other continents (e.g.Craw 1976, Slack and Glime 1985, Suren and Ormerod 1998) revealed high diversity levels and the potential for these organisms to be used in the management of aquatic habitats. This group of plants and their communities are strongly influenced by anthropogenic alterations in natural freshwater ecosystems, with some representatives being recognised as good bioindicators of water quality or the hydromorphological degradation of aquatic habitats (Vanderpoorten and Durwael 1999, Vanderpoorten and Klein 1999a, Gecheva et al. 2010, Ceschin et al. 2012b). Therefore, they have been included, at least in some countries, in the assessment of the ecological status of water bodies for the Water Framework Directive (WFD) as a part of macrophyte vegetation (Gecheva and Yurukova 2014).

Comprehensive floristic studies on a national level, focusing on diversity, distribution, chorology or life-history traits of aquatic and semi-aquatic bryophytes are very scarce. In Europe, floristic studies were mostly focused on a single watercourse or particular river catchment (Papp 1999, Vanderpoorten and Klein 1999b, Papp et al. 2006, Yurukova and Gecheva 2014), while only several studies included larger regions, for example, central Italy (Ceschin et al. 2012a) and north-western Portugal (Vieira et al. 2012a). Moreover, both floristic and ecological studies were largely focused on headwater streams (Papp and Rajczy 1998b, Lang and Murphy 2012, Tremp et al. 2012, Vieira et al. 2014, Ceschin et al. 2015, Vieira et al. 2018) and only seldom included middle and lower river sections (e.g. Papp and Rajczy 1998a, Vanderpoorten and Klein 1999b, Scarlett and O'Hare 2006, Gecheva et al. 2010) or standing waters, in which bryophytes do occur, but are never the dominant part of the vegetation.

Regarding southeast Europe, freshwater bryoflora is significantly better investigated in Bulgaria than in the rest of this region. Several papers dealing with diversity, ecology, as well as the bioindication potential of these species and their communities are available (Papp et al. 2006, Gecheva et al. 2010, Yurukova and Gecheva 2014, Gecheva et al. 2017), while other parts of the region remain under-researched, with only a few studies dealing with aquatic and riparian assemblages from several watercourses in Greece (Papp et al. 1998, Papp 1999). Furthermore, only a few historical publications from the mid-20th century, focusing mainly on the karst river vegetation and the tufa-formation processes, have contributed to knowledge on this otherwise poorly known group in Croatia (e.g. Pavletić 1957, Matoničkin and Pavletić 1961, Pavletić and Matoničkin 1965). In general, the knowledge of the Croatian bryophyte flora is still insufficient and virtually all recent field studies have revealed new national records (e.g. Papp et al. 2013, Sabovljević et al. 2018, Alegro et al. 2019, Rimac et al. 2019a, Rimac et al. 2019b, Ellis et al. 2020, Šegota et al. 2021b, Šegota et al. 2021c, Ellis et al. 2021a, Ellis et al. 2021b). However, some species, regarded as common on a European level, have been recorded in only a few localities in Croatia (Alegro and Šegota 2022), indicating the necessity of further research into the bryophytes.

Given that systematic and comprehensive studies on bryophytes inhabiting freshwater habitats are absent from Croatia, we aimed to:

1. provide the first comprehensive inventory of this understudied group,
2. analyse the distribution and diversity patterns on a national level,
3. examine the chorological spectrum and life-history traits of bryophytes, as well as potential differences between Croatian hydrological and biogeographical regions.

## Material and methods

### Study Area

**Figure 1.**
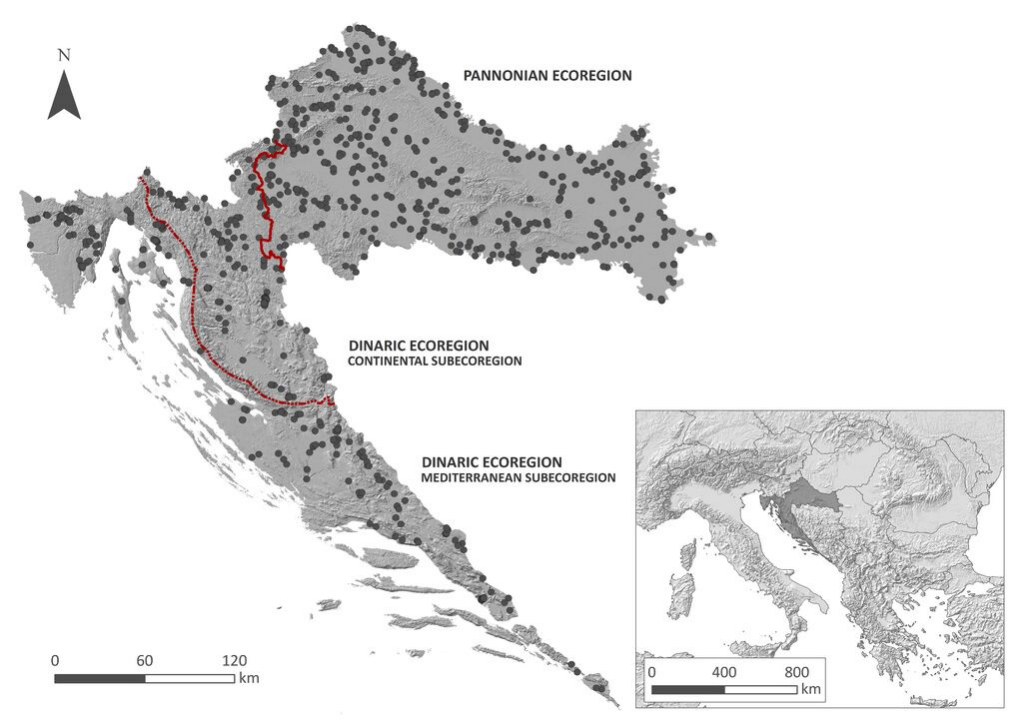
Study area with 786 sampling sites distributed across Croatia (southeast Europe).

**Figure 2.**
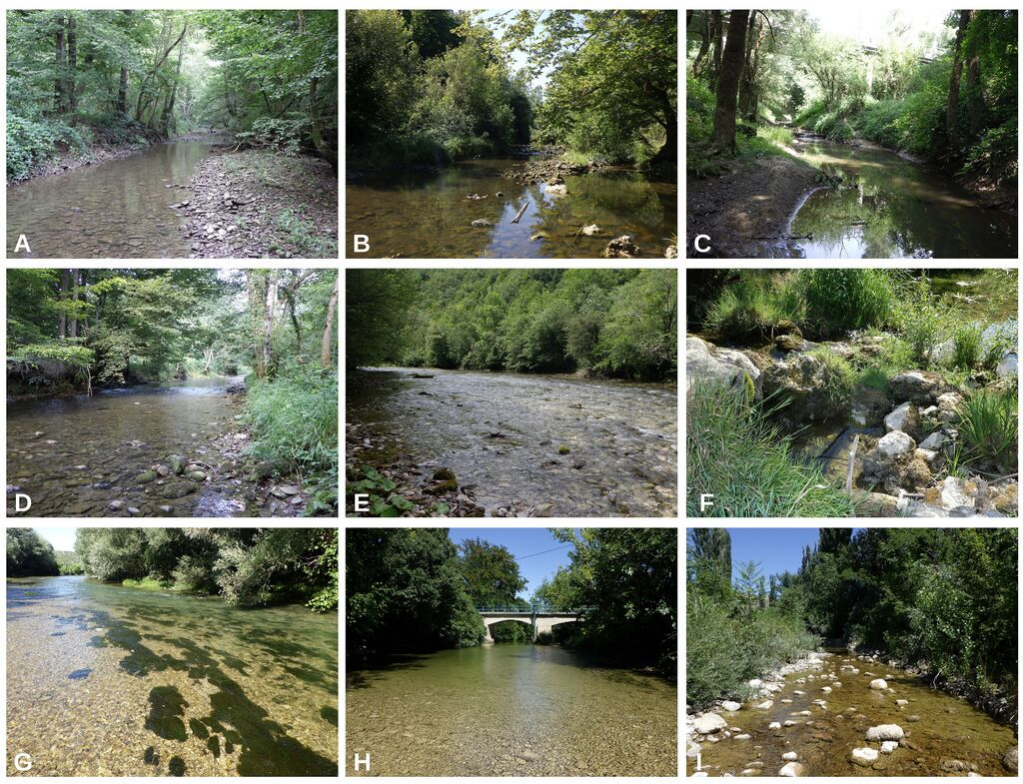
Examples of the sampling sites in Croatia: **Pannonian Ecoregion**: A–Petrinjčica River (Miočinovići), B–Trepča River (Trepča), C–Kravarščica River (Dabići); **Dinaric Ecoregion; Continental Subecoregion**: D–Curak River (at confluence with the Kupa), E–Kupa River (Kupari), F–Korana River (Veljun); **Dinaric Ecoregion; Mediterranean Subecoregion**: G–Krka River (Marasovine), H–Zrmanja River (Butiga), I–Kobilica River (Kusac).

Data on the distribution of bryophytes of freshwater habitats were collected within the national surface water monitoring scheme, i.e. the monitoring of macrophyte vegetation, to assess the ecological status of the water bodies as required by the WFD (European Community 2000). The sampling sites were originally selected so as to encompass the heterogeneity of different water body types recognised by the recent typology developed as a basis for the monitoring of surface waters (Anonymous 2019). According to this typology, the territory of Croatia, of 56,594 km^2^, is divided into two hydrological and biogeographical regions – the Pannonian and the Dinaric Ecoregion, the latter being subdivided into Continental and Mediterranean subecoregions (Fig. 1). A total of 382 watercourses (290 rivers and 92 artificial or heavily modified watercourses) and 45 standing water bodies (nine natural and 36 artificial or heavily modified) were surveyed during the vegetation seasons from 2016 to 2021. The survey included 786 sampling sites (648 on watercourses and 138 on standing waters) ultimately covering the whole of the Croatian territory (Fig. 1). The watercourses were represented by 528 sampling sites on streams and rivers and 120 on artificial and heavily-modified watercourses, while 40 sites were situated on natural lakes and 98 on artificial and heavily-modified standing water bodies (Fig. 2). Each sampling site was visited once during the survey. The altitude of sampling sites ranges from 1 to 711 m a.s.l., with 77.7% of the sampling sites located below 400 m a.s.l.

The Pannonian Ecoregion encompasses the continental part of the country, situated between three large rivers (Sava, Drava and Danube). This area consists of alluvial and diluvial plains with altitudes ranging between 80 and 135 m, along with rather low, solitary mountain massifs. According to lithological and geological composition, most of the Pannonian area belongs to silicate Quaternary deposits, while limestone is found only in the highest mountain areas. The climate is temperate, without a dry season, with warm summers in most of the territory (Cfb) and hot summers predominantly in the eastern part (Cfa) (Beck et al. 2018). The Dinaric Ecoregion is predominantly built of limestone and dolomite bedrock with characteristic karstic phenomena. This ecoregion is characterised by the Dinarides, the largest uninterrupted karst landscape in Europe occupying almost 50% of the territory of Croatia. As the area is, for the most part, built of calcareous and dolomite bedrock, many rivers have partly subterranean courses, flowing through impressive canyons or complex systems of barrage lakes and participating in the karst relief formation. The Continental Subecoregion is characterised by a continental climate (Cfb), while the climate of the Mediterranean Subecoregion is mostly Mediterranean, i.e. temperate with dry and hot summer months (Csa) (Beck et al. 2018). The Pannonian watercourses belong exclusively to the Black Sea Basin, as do the majority of the watercourses of the Dinaric-Continental Subecoregion. The watercourses of the Dinaric-Mediterranean Subecoregion, on the other hand, belong to the Adriatic Sea Basin. The estimated total length of natural and artificial watercourses in Croatia is 32,100 km (Biondić 2009), while there are only a dozen fairly large natural lakes in the country.

### Sampling Method

A survey of macrophyte vegetation was performed according to the national methodology for macrophyte sampling (Anonymous 2019) from 2016 to 2021, from June to September when macrophyte vegetation is optimally developed and during the lowest water discharge levels. Watercourses were surveyed for macrophytes along 100 m-long transects, while 6×100 m transects were used when surveying macrophytes in lakes. The riverbeds were inspected for bryophytes from the banks and, if the water depth was low enough, by zigzagging across the channel. Standing waters were sampled by boat and additionally by walking along the banks. In less-accessible areas, the river/lake bottom was raked to reach the macrophytes, with the rake either on a long pole or at the end of a rope. Species coverage and abundance were assessed using the extended Braun-Blanquet scale (r = one individual, + = up to 5 individuals, 1 = up to 50 individuals, 2m = over 50 individuals, 2a = coverage 5–15%, 2b = coverage 15–25%, 3 = 25–50%; 4 = coverage 50–75%; 5 = coverage over 75%) (Barkmann et al. 1964, Braun-Blanquet 1964, Dierschke 1994). These classes were transformed into modified classes, representing the mean cover values of Braun-Blanquet classes (Tremp 2005), in order to calculate the species’ average covers. Bryophytes were collected from various substrates (rocks, boulders, pebbles, woody debris, silt) within the riverbed, as well as from marginal submerged tree stumps and periodically flooded margin slopes (drawdown zone). The collected material was deposited in herbarium ZA (Thiers 2022). The nomenclature follows Hodgetts et al. (2020) and Erzberger and Schröder (2013) for *Bryumbarnesii*.

### Analysis

The chorological analysis of bryophyte flora was carried out according toHill and Preston (1998), who divided floristic elements into categories with similar climatic requirements. The basis of this method is a two-dimensional grid, reflecting: 1) major biomes, which are combinations of zonobiomes (latitudinal zones) and the equivalent orobiomes (zones on mountains) and 2) the eastern limits in Eurasia. The analysis of life-form spectra was done using the classification given in Hill et al. (2007), while the life strategies were defined according to During (1992) given in Dierßen (2001). For a few species that were not listed in these classifications, we assigned one of the categories based on the known distribution of the particular species in case of the chorotypes and morphologically and ecologically similar species in case of life-forms and life strategies. The species’ affinity to water, i.e. different freshwater microhabitats in relation to humidity level, was analysed using the classifications given by Hill et al. (2007), Dierßen (2001) and Vitt and Glime (1984). The species’ threat status follows Hodgetts et al. (2019). Margalef and Shannon-Wiener alpha diversity indices were calculated and presented through boxplots using Past 4.5 software (Hammer et al. 2001). The altitude was obtained from digital elevation model of 5×5 m resolution, while CHELSA climatological datasets (Karger et al. 2017) were used to describe the climatological conditions. Distribution maps were created using ArcGIS 10.5 software.

## Results

**Figure 3.**
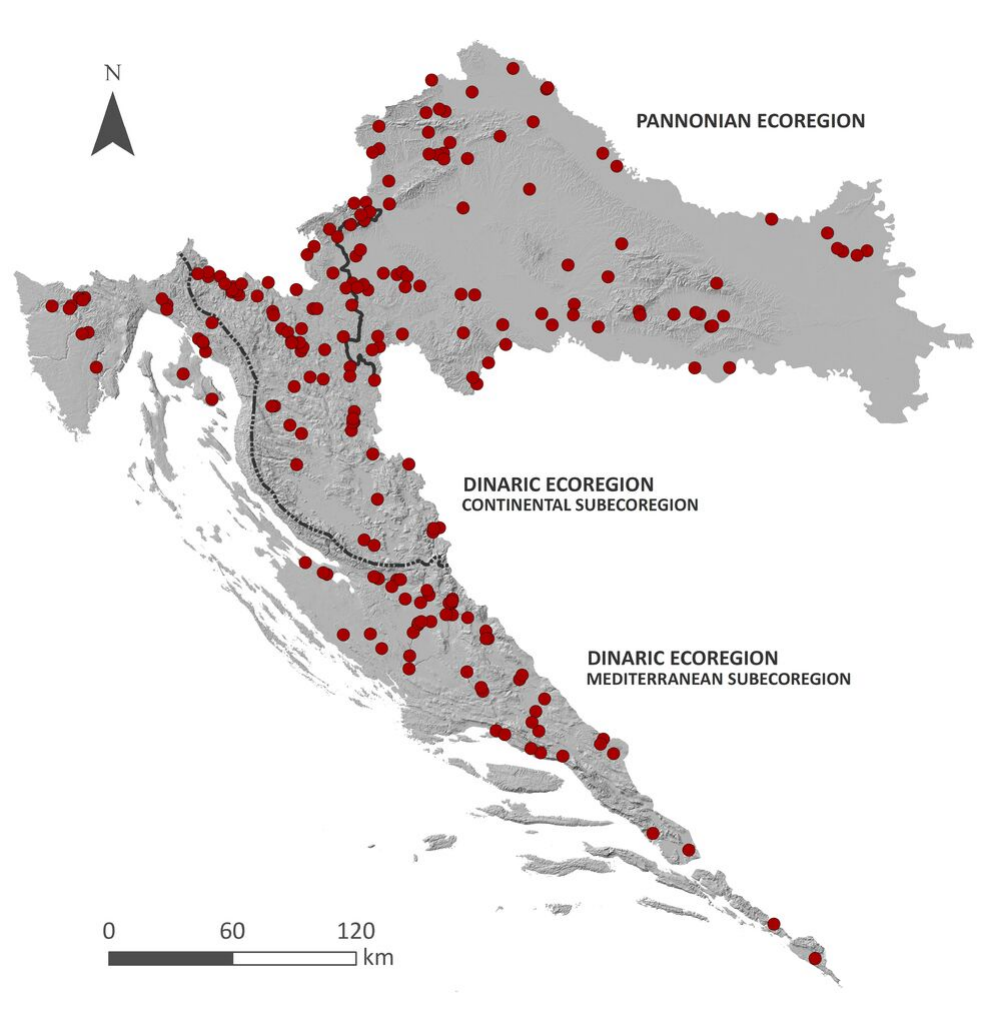
Distribution of 228 sampling sites with freshwater bryophytes in Croatia.

Aquatic and semi-aquatic bryophytes were present at 228 (29%) of the sampling sites (Fig. 3). The sites with bryophytes were distributed on 160 (38%) of the surveyed water bodies, i.e. on 140 (37%) surveyed watercourses and 20 (45%) surveyed standing water bodies.

**Figure 4.**
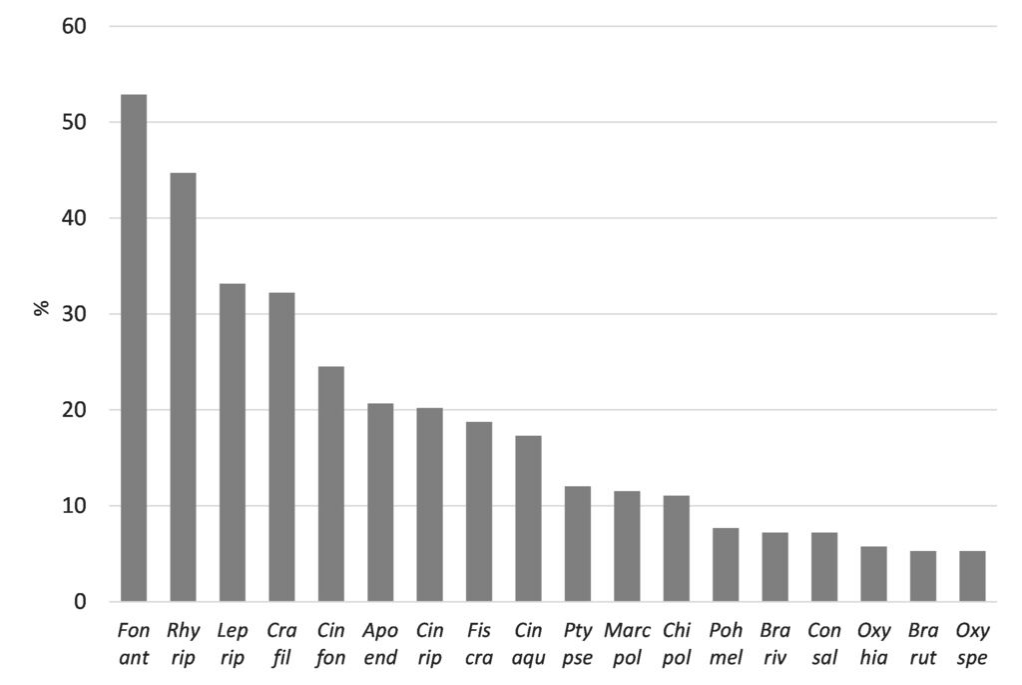
The most frequent bryophyte species (only species present in over 10 sampling sites are shown) (Font ant–*Fontinalisantipyretica*, Rhy rip–*Rhynchostegiumriparioides*, Lep rip–*Leptodictyumriparium*, Cra fil–*Cratoneuronfilicinum*, Cin fon–*Cinclidotusfontinaloides*, Apo end–*Apopelliaendiviifolia*, Cin rip–*Cinclidotusriparius*, Fis cra–*Fissidenscrassipes*, Cin aqu­–*Cinclidotusaquaticus*, Pty pse–*Ptychostomumpseudotriquetrum*, Marc pol–*Marchantiapolymorpha*, Chi pol–*Chiloscyphuspolyanthos*, Poh mel–*Pohliamelanodon*, Bra riv–*Brachytheciumrivulare*, Con sal–*Conocephalumsalebrosum*, Oxy hia–*Oxyrrhynchiumhians*, Bra rut–*Brachytheciumrutabulum*, Oxy spe–*Oxyrrhynchiumspeciosum*).

**Figure 5.**
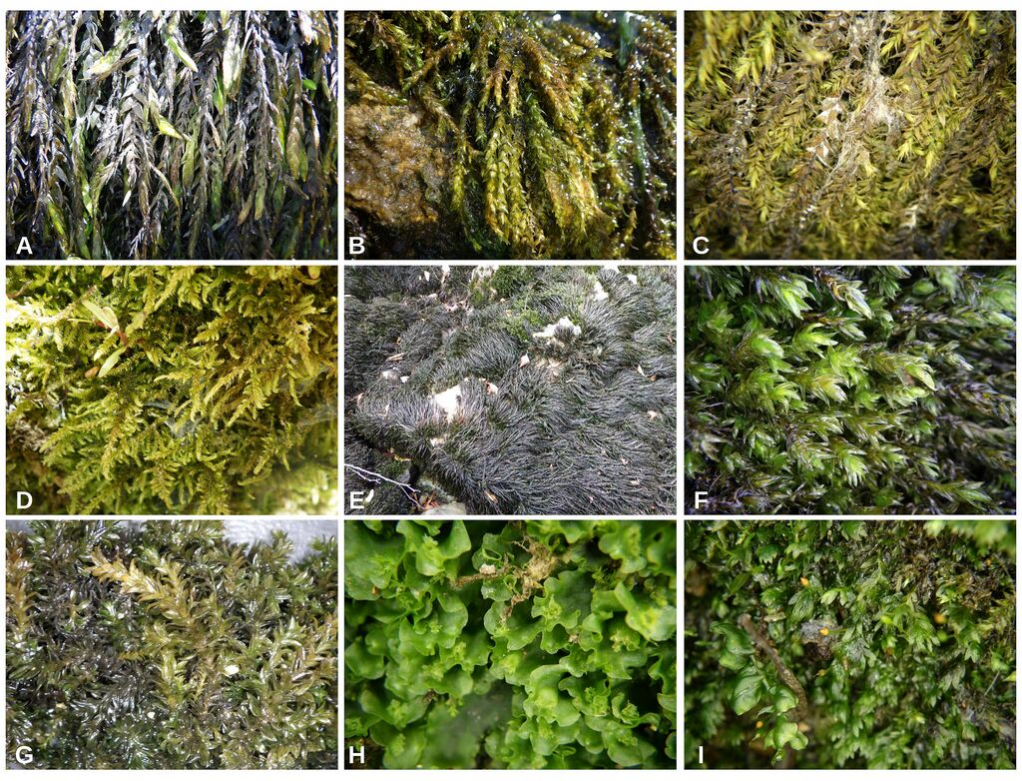
The most common freshwater bryophyte species in Croatia: A–*Fontinalisantipyretica*, B– *Rhynchostegiumriparioides*, C–*Leptodyctiumriparium*, D–*Cratoneuronfilicinum*, E­–*Cinclidotusaquaticus*, F­–*C.fontinaloides*, G–*C.riparius*, H–*Apopelliaendiviifolia*, I–*Fissidenscrassipes*.

Eighty-three bryophyte species, including 68 mosses (Bryophyta) and 15 liverworts (Marchantiophyta), were recorded (Table 1,Suppl. material 1). Mosses were represented by 43 acrocarpous and 25 pleurocarpous species, while liverworts included four leafy and 11 thalloid species. The most frequent species, found at as many as 53% of sampling sites, was *Fontinalisantipyretica*, followed by *Rhynchostegiumriparioides* (45%), *Leptodictyumriparium* (33%) and *Cratoneuronfilicinum* (32%) (Figs 4, 5). Amongst liverworts, the most common species were *Apopelliaendiviifolia* (21%), *Marchantiapolymorpha* (12%), *Chiloscyphuspolyanthos* (11%) and *Conocephalumsalebrosum* (7%). The majority of the 83 recorded species were rarely found. Over 40% of the species were registered at a maximum of three sampling sites, while over 70% of species were found on less than 10 sampling sites (Table 1).

**Figure 6.**
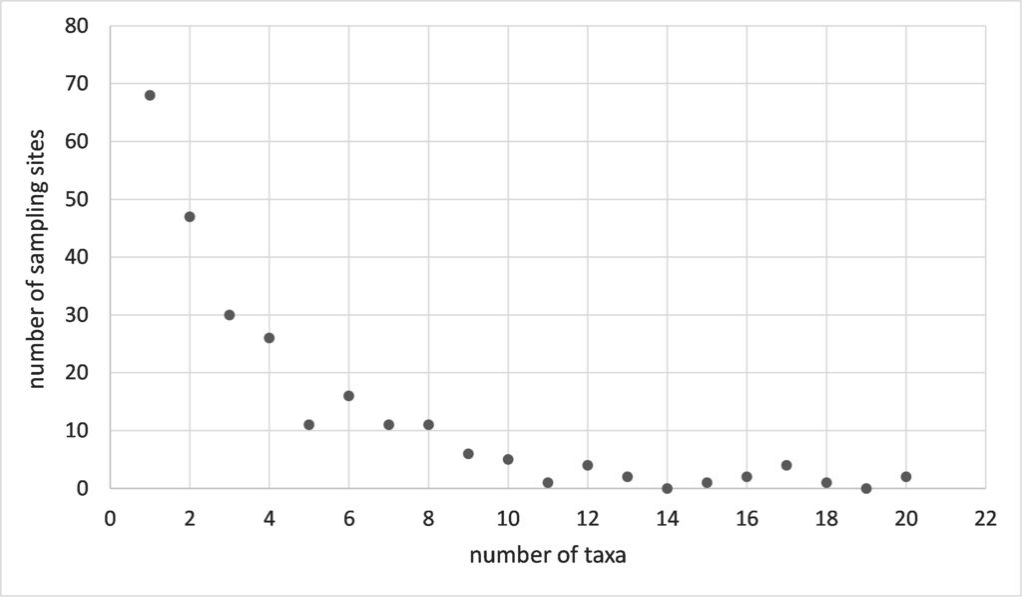
The number of species per sampling site.

The overall average bryophyte species richness at the 228 sites was 4.17 ± 0.25 species per site, while 27% of the sites had only one species and other sites up to a maximum of 20 species (Fig. 6). Two-thirds of the sampling sites in the present study were species-poor (containing fewer than four species) and one third were species-rich (with more than four species).

**Figure 7.**
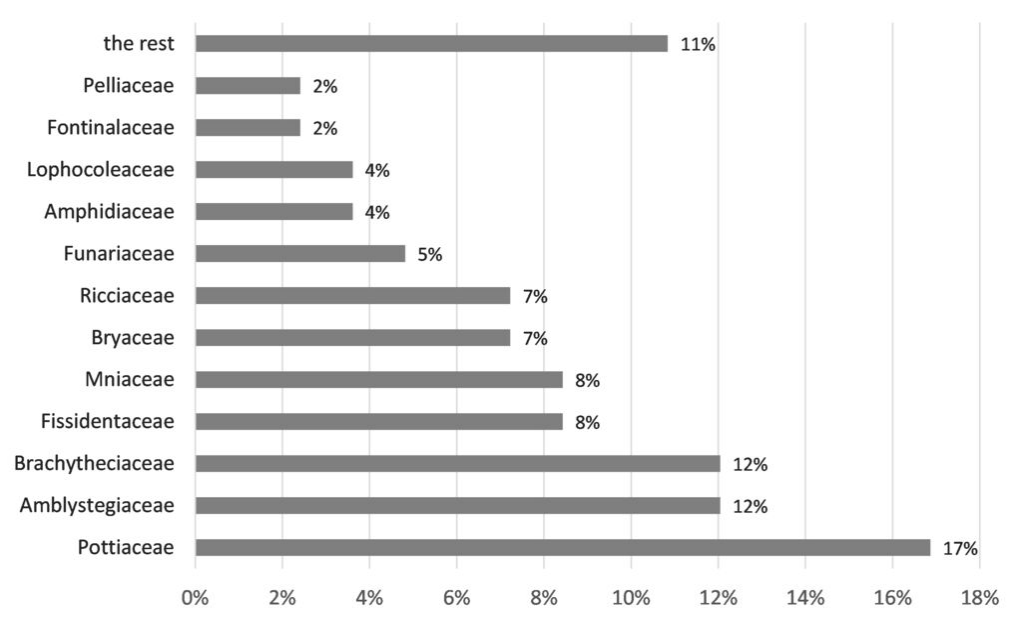
The most represented families of freshwater bryophytes in Croatia.

The collected species belong to 10 orders, 21 families and 43 genera (Table 1). Regarding the number of recorded species, the families most represented were Pottiaceae (14), Amblystegiaceae and Brachytheciaceae (10 each), Fissidentaceae and Mniaceae (seven each), Bryaceae and Ricciaceae (six each) (Fig. 7). Genera with the highest number of recorded species were *Fissidens* (seven species) and *Bryum*, *Didymodon* and *Riccia* (five species each) (Table 1).

The vast majority of recorded species had quite low coverage in survey localities, with the mean coverage of all species being 3.3%. As many as 69 species had a mean cover in the investigated sites of less than 5%, whereas just three species displayed a mean coverage greater than 10% (*Hymenostyliumrecurvirostrum* – 22.5%, *Palustriellacommutata* – 17.9% and *Cinclidotusaquaticus* – 17.1%).

The chorological analyses, based on major biomes, indicated the predominance of the temperate chorotype in Croatian freshwater bryoflora: temperate (30.1%), boreo-temperate (24.1%), southern-temperate (21.7%) and wide-temperate (8.4%). The biogeographical spectrum, based on the eastern limit, showed that the dominant chorotypes were circumpolar (54.2%) and European (31.3%). Analysis of life-forms, based on the species frequencies, revealed that the most dominant were aquatic trailings (28%), turfs (18%), rough mats (15%), smooth mats (11%) and wefts (11%). Regarding the life strategy, the most frequent were perennials (34%), colonists (30%) and competitive perennials (19%).

The recorded bryoflora displays rather wide niche heterogeneity concerning the humidity levels preferred. Only six recorded species could be classified as obligate aquatics, having little or no tolerance to drought conditions (*Fissidensarnoldii*, *F.fontanus*, *Fontinalisantipyretica*, F.hypnoidesvar.duriaei, *Hygroamblystegiumfluviatile* and *Ricciocarposnatans*), while the majority (40 species) were facultative aquatics, having some degree of tolerance to desiccation and xerophytic conditions. Seven of the recorded species were semi-aquatic emergents, thriving on a periodically waterlogged substrate. Twenty-five recorded species were associated with moist or moderately moist substrates, whereas the least represented group with only five species was that characteristic of a well-drained terrestrial substrate.

Concerning the threat status, the majority of the recorded species are considered to be of least concern (LC). *Philonotismarchica* is evaluated as endangered (EN), while *Fissidensarnoldii*, *Physcomitriumeurystomum* and *Ph.sphaericum* are vulnerable (VU) species on a European level.

### Dinaric vs. Pannonian Ecoregion

The study revealed that the Dinaric Ecoregion supports higher freshwater bryophyte diversity (70 bryophyte species, out of which 60 are mosses), than the Pannonian Ecoregion with 57 recorded bryophyte species (44 mosses) (Table 2). In contrast, both ecoregions were shown to harbour the same liverwort diversity in aquatic and semi-aquatic habitats, represented by four leafy and 11 thallose species. The two regions share as many as 44 species (53.0%), while 26 species (31.3%) were exclusively found in the Dinaric and 13 species (15.7%) in the Pannonian Ecoregion. In the Dinaric Ecoregion, the dominant species with occurrence frequencies higher than 30%, were *Fontinalisantipyretica*, *Rhynchostegiumriparioides* and *Cinclidotusfontinaloides*, whereas in the Pannonian Ecoregion, the only truly dominant species was *Leptodictyumriparium*. The most common species occurring in both ecoregions were *Fontinalisantipyretica*, *Cratoneurumfilicinum*, *Fissidenscrassipes* and *Marchantiapolymorpha*. The common species in the Dinaric Ecoregion were also *Cinclidotusfontinaloides*, *Apopelliaendiviifolia*, *Cinclidotusaquaticus*, *Ptychostomumpseudotriquetrum*, *Didymodontophaceus* and *Eucladiumverticillatum*, while in the Pannonian Ecoregion *Pohliamelanodon*, *Conocephalumsalebrosum*, *Oxyrrhynchiumhians*, *Peelianeesiana*, *Physcomitriumpatens*, *Oxyrrhynchiumspeciosum* and *Ricciafluitans* were frequent.

The Dinaric Ecoregion had a higher species richness (4.6±0.33 species) and mean coverage (3.6%) per sampling site than the Pannonian Ecoregion (3.4±0.35 species; mean coverage 2.4%) per sampling site (Table 2). In the Dinaric Ecoregion, the dominant bryophyte families were Pottiaceae, Brachytheciace, Amblystegiaceae, Fissidentaceae and Mniaceae, while in the Pannonian Ecoregion, they were Amblystegiaceae, Brachytheciace, Ricciaceae, Fissidentaceae and Mniaceae.

**Figure 8.**
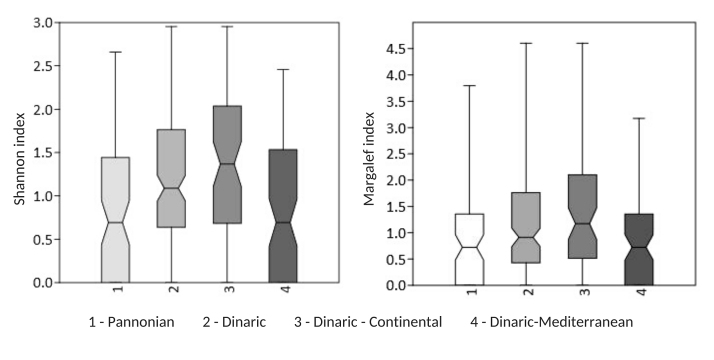
Comparison of alpha diversity (Shannon-Wiener and Margalef alpha diversity indices) in the Pannonian Ecoregion, Dinaric Ecoregion, Dinaric–Continental Subecoregion and Dinaric–Mediterranean Subecoregion.

Within the Dinaric Ecoregion, the Continental Subecoregion showed a higher species richness (65 bryophytes; 55 mosses and 10 liverworts) than the Mediterranean Subecoregion with 40 bryophyte species (33 mosses and seven liverworts) recorded within this study. Furthermore, the Continental Subecoregion features higher species richness (5.9 ± 0.58 species) per sampling site than the Mediterranean Subecoregion (3.4±0.29 species), while the mean coverage per sampling site was similar in both subecoregions (Table 2). The same trends are detectable from the Shannon-Wiener and Margalef alpha diversity indices (Fig. 8).

**Figure 9.**
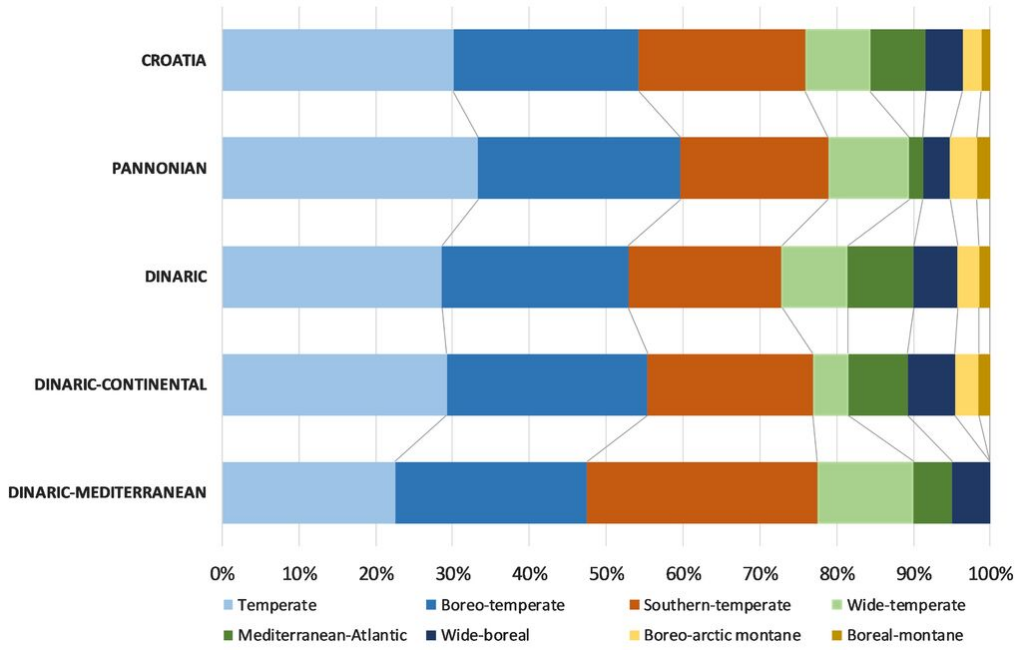
Chorological spectra of freshwater bryophytes, based on major biomes for Croatia, Pannonian Ecoregion, Dinaric Ecoregion, Dinaric–Continental Subecoregion and Dinaric–Mediterranean Subecoregion.

The chorological comparison of Croatian eco- and subecoregions, based on major biomes, revealed large chorotype overlapping, with the dominance of temperate chorotypes; however, some biogeographical differences were highlighted. The Mediterranean-Atlantic chorotype was almost completely absent from the Pannonian Ecoregion, while within the Dinaric Ecoregion, this type was more frequent in the Continental Subecoregion. On the other hand, the boreo-arctic and boreal-montane chorotypes were absent in the Mediterranean Subecoregion (Fig. 9).

**Figure 10.**
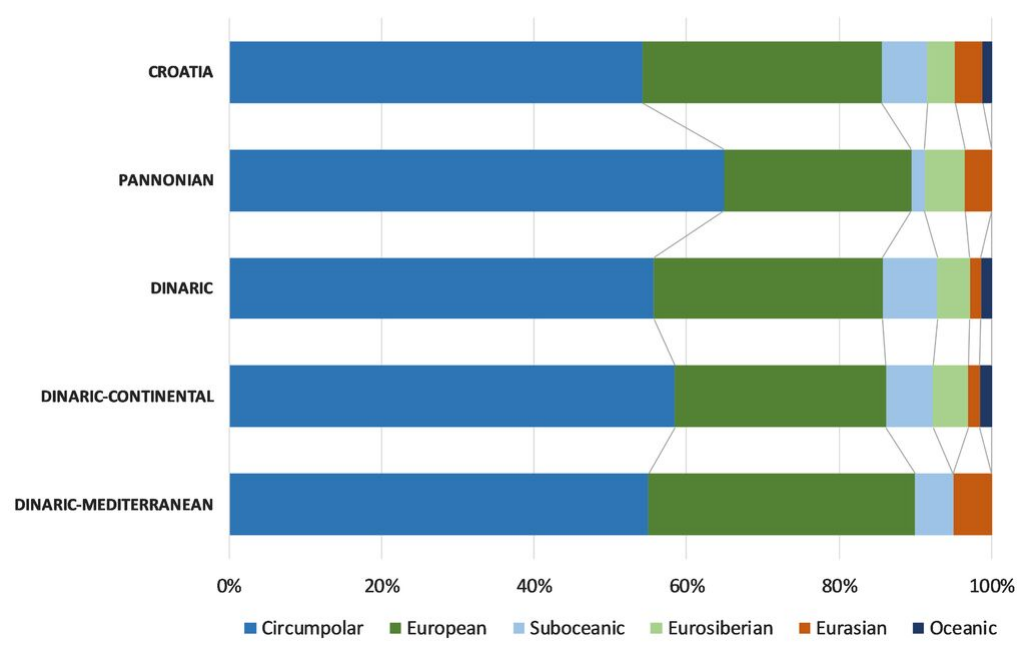
Chorological spectra of freshwater bryophytes, based on the eastern limit for Croatia, Pannonian Ecoregion, Dinaric Ecoregion, Dinaric–Continental Subecoregion and Dinaric–Mediterranean Subecoregion.

The chorological comparison of Croatian eco- and subecoregions, based on the eastern limit, showed the dominance of circumpolar and European chorotypes in all eco- and subecoregions (Fig. 10). The sub-oceanic and oceanic chorotypes are very rare in the Pannonian Ecoregion, while in the Dinaric Ecoregion they are more common in the Continental Subecoregion.

**Figure 11.**
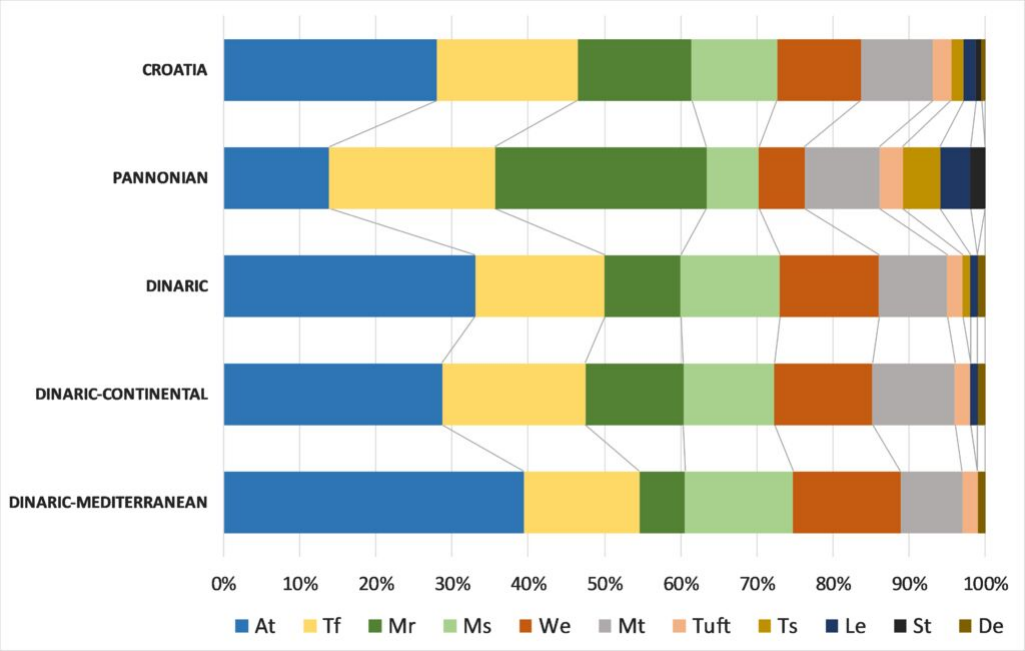
Life-form spectra of freshwater bryophytes for Croatia, Pannonian Ecoregion, Dinaric Ecoregion, Dinaric–Continental Subecoregion and Dinaric–Mediterranean Subecoregion, based on species frequencies (At–Aquatic trailing, De–dendroid, Le– lemnoid, Mr–rough mat, Ms–smooth mat, Mt–thalloid mat, St–solitary thalloid, Tf–turf, Ts–scattered turf, Tuft–tuft and We–weft).

Bryophyte life-forms were not evenly distributed within Croatian eco- and subecoregions (Fig. 11), with the most conspicuous difference in the share of the aquatic trailings. In the Dinaric Ecoregion, this life-form predominates (33%) and, in the Pannonian, it reaches only 14% considering the frequency of the species with that particular life-form. By contrast, rough mats are almost three times as frequent in the Pannonian (28%) as in the Dinaric Ecoregion (10%). Finally, wefts are twice as frequent in the Dinaric (13%) as in the Pannonian Ecoregion (6%).

**Figure 12.**
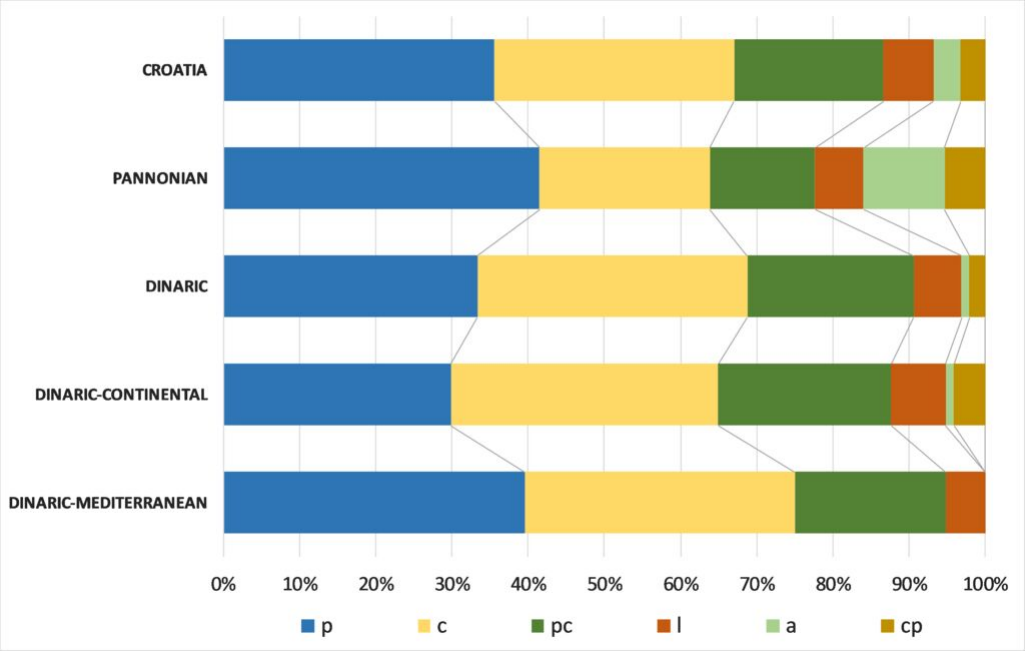
Life strategy spectra of freshwater bryophytes for Croatia, Pannonian Ecoregion, Dinaric Ecoregion, Dinaric–Continental Subecoregion and Dinaric–Mediterranean Subecoregion, based on species frequencies (p–perennials, c–colonists, pc–competitive perennials, l–long-lived shuttle, a–annual shuttle, cp–pioneer colonists).

Regarding the life strategies, all Croatian eco- and subecoregions feature the dominance of perennial and colonist bryophyte species. However, competitive perennial strategy is almost twice as frequent in the Dinaric Ecoregion (21%) as in the Pannonian (13%). Contrarily, the annual shuttle strategy in the Dinaric Ecoregion is almost negligible (1%), while in the Pannonian Ecoregion, it is relatively more frequent (10%) (Fig. 12).

## Discussion

The present study is the first to compile a comprehensive floristic catalogue on Croatian freshwater bryophyte species including 83 species representing 12% of Croatian bryoflora (Alegro and Šegota 2022). Mosses were represented with notably higher number of species than liverworts, as already reported in previous work focusing on the freshwater bryoflora (Muotka and Virtanen 1995, Scarlett and O'Hare 2006, Gecheva et al. 2010, Ceschin et al. 2012a, Vieira et al. 2012a), which was to be expected given their low resistance to mechanical water scouring and desiccation, as well as continuous submersion compared to mosses (Gimingham and Birse 1957, Kimmerer and Allen 1982, Vitt and Glime 1984). Furthermore, they also appear more sensitive to changes in catchment land use and to elevated stream nutrient levels (Suren 1996). While the foliose liverwort *Chiloscyphuspolyanthos* was the only quite frequent liverwort in fast-flowing streams, thallose species were common in splash zones and margins of rivers (e.g. *Conocephalumsalebrosum*, *Lunulariacruciata*, *Pellianeesiana*, *A.endiviifolia*, *Marchantiapolymorpha*) in our study.

The majority of species encountered are not considered to be truly aquatic, confirming other studies investigating the bryoflora of streams and rivers, for example, in the UK (Scarlett and O'Hare 2006), Portugal (Vieira et al. 2012a), Bulgaria (Gecheva et al. 2010) and Italy (Ceschin et al. 2012a). These studies, like our own, included both species growing permanently submerged in the riverbed, as well as those on riverbanks and other associated periodically submerged microhabitats. In our study, only six species (*Fissidensarnoldii*, *F.fontanus*, *Fontinalisantipyretica*, F.hypnoidesvar.duriaei, *Hygroamblystegiumfluviatile* and *Ricciocarposnatans*) were considered obligate aquatics *sensu* Vitt and Glime (1984) or rheophilic or limnophylic *sensu Dierßen (2001)*, living regularly submerged in running waters or on the surface of standing water (Hill et al. 2007) and having little or no tolerance to drought conditions and desiccation. About half the species were facultative or semi-aquatics *sensu* Vitt and Glime (1984) or hydrophytic to hygrophytic *sensu Dierßen (2001)*. This was expected, since in general, only a few bryophyte species are considered truly aquatic and, additionally, obligate aquatics are more characteristic of limnophilous habitats (Vitt and Glime 1984), which were less represented in this study. By contrast, the more numerous facultative aquatics are better adapted to rheophilous environments (Vitt and Glime 1984), which were dominant in our study. The rest of the species can be described as mesophytic to hygrophytic, living on a moderately wet substrate, adapted to some degree of xerophytic conditions. They are mostly terricolous species that have found an alternative niche in riparian microhabitats.

As previously recorded in other studies (e.g. Ceschin et al. 2015), most of the freshwater bryophyte species show both low occurrences and low cover with respect to the sampled area. Species richness was lower in the Pannonian Ecoregion (3.4), while in the Dinaric, it was 4.6, which corresponds well with the species richness of highly seasonal Mediterranean rivers (4.8 species per site) (Vieira et al. 2018). The Continental part of the Dinaric Ecoregion harbours the highest species richness per site in Croatia (5.9) which is related to the very good ecological status of watercourses, with clear, cold, well-oxygenated and fast-flowing water, as well as rocky substrates (Mihaljević et al. 2020). This subecoregion largely corresponds to Mediterranean Mountains according to the European Environmental Stratification (Metzger et al. 2005), while the rest is in the Alpine Region. The average species richness of freshwater bryophytes for Mediterranean Mountains was estimated at 4.5 (Vieira et al. 2018), being somewhat lower than that of the Continental Subecoregion of Croatia.

The lowest diversity was observed in the Pannonian Ecoregion, which is presumably related to the dominant characteristics of the water bodies there. These are mostly slow, eutrophic lowland streams and rivers with unstable sandy and gravelly alluvial sediments and higher depth of the water column (Mihaljević et al. 2020). Moreover, the freshwater bryophytes here are subject to intense competition with vascular macrophytes, leading to an overall lower coverage and richness or the complete absence of bryophytes (Vitt and Glime 1984, Glime 1992). Furthermore, the majority of watercourses in the Pannonian Ecoregion are subjected to a significant level of hydromorphological alterations, such as flow regulation through canalisation, riverbed deepening and embankment, as well as considerable changes in land-use practice, with riparian vegetation being removed (Vučković et al. 2021), while nutrient input and water pollution are increasing substantially, thereby reducing the habitat quality for bryophytes. Bryophytes are generally absent from streams flowing through modified catchments of easily eroded geology or small substrate sizes and shallow gradients. These streams may also have relatively high nutrient levels affecting the bryophyte cover and communities (Suren 1996, Gecheva et al. 2010, Ceschin et al. 2012b, Gecheva et al. 2017).

The low mean coverages of all species (3.3%) can be explained by the fact that our study included evenly upper, middle and lower river sections. In our study, only three species with mean coverage greater than 10% were either tufa-forming mosses of waterfalls, such as *Hymenostyliumrecurvirostrum* and *Palustriellacommutata* (Mucina et al. 2016, Lyons and Kelly 2017) or mosses characteristic of headwater streams as *Cinclidotusaquaticus* (Kochjarová et al. 2007, Ceschin et al. 2012b). In such habitats, the bryophytes form large colonies, having no competition from vascular plants which are not able to withstand such harsh environments, i.e. cold, fast-flowing water and rocky substrates (Suren 1996, Tremp et al. 2012). Additionally, bryophytes have lower demand for nutrients which allows them to thrive in headwater streams, characterised by low nutrient levels (Vanderpoorten and Goffinet 2009).

*Fontinalisantipyretica* and *Rhynchostegiumriparioides*, the most abundant and common aquatic species in our study, were also amongst the most frequent aquatic species in other surveys (Scarlett and O'Hare 2006, Gecheva et al. 2010, Ceschin et al. 2012a, Ceschin et al. 2015). The occurrence of *F.antipyretica* was not previously related to specific physico-chemical and trophic conditions, suggesting a wide ecological behaviour (Muotka and Virtanen 1995, Vanderpoorten et al. 1999, Scarlett and O'Hare 2006, Ceschin et al. 2012b). On the other hand, different studies gave contradictory results regarding the ecological preferences of *R.riparioides*, most of them referring to this species as acid sensitive and characteristic of unpolluted running waters (Ceschin et al. 2012b, Tremp et al. 2012). While *F.antipyretica* was present in both Croatian ecoregions, both in watercourses and standing waters, *Rhynchostegiumriparioides* was more frequently found in the Dinaric Ecoregion, while in the Pannonian Ecoregion, it was mostly restricted to smaller and faster streams. The only dominant species in the Pannonian Ecoregion was found to be *Leptodictyumriparium*, which has already been detected as the most abundant and characteristic species of middle and lower stream sections (Papp et al. 2006, Gecheva et al. 2010, Ceschin et al. 2012b) and is regarded as the most pollution-tolerant (Frahm 1974), with preferences for eutrophic waters (Vanderpoorten et al. 1999, Ceschin et al. 2012b).

Amongst dominant families, Amblystegiaceae, Brachytheciaceae, Fissidentaceae and Mniaceae were common in both ecoregions. However, the most represented family in the Dinaric Ecoregion was Pottiaceae, reflecting the presence of karstic watercourses in this region, a suitable habitat of aquatic species within the genus *Cinclidotus*, the tufa-forming *Didymodontophaceus* and several other *Didymodon* species inhabiting the periodically submerged niches and splashing zones along karstic rivers. By contrast, in the Pannonian Ecoregion, the family *Ricciaceae* is the third most represented family, with free-floating *Ricciafluitans*, *R.rhenana* and *Ricciocarposnatans*, characteristic of stagnant and slow-flowing lowland streams or canals with eutrophic water and several other *Riccia* species recorded on fine gravelly and sandy drawdown zones of the watercourses and standing water bodies in the Croatian lowlands. The species exclusive to the Dinaric Ecoregion (*Didymodoninsulanus*, *D.spadiceus*, *Hygrohypnumluridum*, *Hymenostyliumrecurvirostrum*, *Philonotismarchica*, *Rhynchostegiellacurviseta*, *R.teneriffae* etc.) were associated with stable rocky substrates, cold, clear, well-oxygenated waters of karstic rivers and their springs, characteristic of this region (Mihaljević et al. 2020). The species restricted to the Pannonian Ecoregion (*Leptobryumpyriforme*, *Pellianeesiana*, *Physcomitriumeurystomum*, *Ph.sphaericum*, *Ricciafrostii*, *R.glauca*, *R.rhenana*, *Ricciocarposnatans*, *Fissidenspusillus* etc.) were associated with moist and fine-textured substrata of the margins of lakes, reservoirs and rivers, with the exception of *F.pusillus*, a saxicolous species which was found in semi-mountain springs with a siliceous bedrock.

Considering the chorological spectrum of studied flora, the prevailing presence of temperate (circumpolar) species corresponds with the biogeographical characteristics of the studied area. This was also detected in the bryoflora of running waters of central Italy (Ceschin et al. 2012a) and the European Mediterranean Region (Vieira et al. 2018). The chorological comparison of Croatian sub- and ecoregions revealed some biogeographical peculiarities. The rarity of Mediterranean-Atlantic, as well as of suboceanic and oceanic chorotypes (e.g. *Rhynchostegiellacurviseta*, *R.teneriffae*, *Lunulariacruciata*) in the Pannonian Ecoregion and the presence of those in the Dinaric–Continental Subecoregion largely corresponds with the climatic limitations. The mean air temperatures of the wettest quarter are significantly higher in the Pannonian (17.4±0.4°C) than in the Dinaric Ecoregion (11.1±0.2°C) and mean air temperatures of the driest quarter are significantly lower in the Pannonian (3.3±0.1°C) than in the Dinaric Ecoregion (14.1±0.8°C) (Karger et al. 2017). Moreover, the amount of precipitation is significantly lower in the Pannonian Ecoregion and highest in the Dinaric–Continental Subecoregion (Table 2). Similarly, the absence of boreo-arctic and boreal-montane chorotypes (e.g. *Dichodontiumflavescens*, *D.pellucidum*, *Plagiomniumellipticum*) in the Dinaric-Mediterranean Subecoregion is most likely conditioned by the higher mean annual air temperature and the annual precipitation amount in this region (Table 2).

Bryophyte life-forms can be interpreted as recurring arrangements of the photosynthetic tissues that minimise evaporative water loss and maximise primary production (Bates 1998). Life-forms of aquatic bryophytes present better adaptations to seasonal desiccation and dragging forces either during permanent submersion or flood events, with a firmer structure able to resist mechanical forces (Vitt and Glime 1984, Muotka and Virtanen 1995, Fritz et al. 2009). The dominant life-form in our study were aquatic trailings, described as aquatic bryophytes (mostly mosses) attached to the substrate and trailing in the water (Hill et al. 2007). They correspond with “streamers”, a term defined by *Glime (1968)* and used in Vieira et al. (2012b), which includes long, dangling aquatics (e.g. *Chiloscyphuspolyanthos*, *Cinclidotusaquaticus*, *C.fontinaloides*, *C.riparius*, *Fissidensfontanus*, *Fontinalisantipyretica*, F.hypnoidesvar.duriaei). They are associated with more deeply submerged sites (found up to 30 cm of depth), mostly in the slower currents of streambed in full sunlight (Vieira et al. 2012b). Turfs, the second most represented life-form, feature many loosely or closely packed vertical stems with limited branching (Bates 1998, Hill et al. 2007). They colonise microhabitats usually subjected to seasonal floods with a strong impact of water (e.g. *Ptychostomumpseudotriquetrum*, *Dichodontiumflavescens*, *Didymodontophaceus*, *Fissidenscrassipes*, *Hymenostyliumrecurvirostrum*, *Philonotismarchica* etc.); however, they are not very hydrodynamic-resistant, both to desiccation and water abrasion (Vitt and Glime 1984, Vieira et al. 2012b). When the ecoregions are compared, the life-form spectra show considerable differences. While in the Dinaric Ecoregion, aquatic trailings associated with fast-flowing karst streams prevail (e.g. *Cinclidotus* spp.) (33%), in the Pannonian Ecoregion, a similar proportion is displayed by the rough mats category, represented by aquatic species (dominant *Leptodictyumriparium* and the quite rarely recorded *Hygroamblystegiumfluviatile* and *H.tenax*) and species mostly found inhabiting riparian zones of the shaded lowland forest streams and rivulets (*Brachytheciummildeanum*, *B.rivulare*, *B.rutabulum*, *Oxyrrhynchiumhians*, *O.speciosum*).

Amongst Croatian freshwater bryophytes, the most frequent are those with a potential lifespan longer than one year. This includes perennial life strategy (*Fontinalis*, *Palustriella*, *Brachytecium*, *Hygroamlystegium* etc.) and several-year life-span colonists (*Ptychostomum*, *Cinclidotus*, *Dichodontium*, *Didymodon*, *Fissidens*, *Apopeelia* etc.). This is concurrent with the fact that aquatic species are mostly perennial, pleurocarpous mosses (Glime 2020) and that submersed bryophyte communities are mostly characterised by perennials and ephemeral colonists (Vieira et al. 2012b). In general, perennials are more likely to be found in permanent fast-flowing currents, whereas colonists are more common in the lower currents or emergent positions (Glime 2020). In the Dinaric Ecoregion, the competitive perennial strategy is twice as frequent as in the Pannonian, mainly because of the high frequencies of species associated with karstic streams and tufa formations, (e.g. *Ptychostomumpseudotriquetrum*, *Calliergonellacuspidata*, *Chiloscyphuspallescens*, *Ch.polyanthos*, *Cratoneuronfilicinum*, *Palustriellacommutata*, *P.falcata*) which are absent from lowland Pannonian watercourses. On the contrary, the Pannonian Ecoregion shows a ten times higher frequency of annual shuttle life strategy than the Dinaric Ecoregion. Annual shuttle species (*Physcomitriumeurystomum*, *Ph.patens*, *Ph.sphaericum*, *Ricciacavernosa*, *R.fluitans*, *R.frostii*, *R.glauca*, *R.rhenana*, *Ricciocarposnatans*) are short-lived species with high reproductive effort, i.e. producing numerous spores (During 1979, Kürschner 2004). These ephemeral terricolous species are successful on the margins of lowland slow-flowing or stagnant waters, where they can germinate on deposited, fine-textured sediments and finish their whole life cycle within a brief period when water withdraws from gently sloping margins. This period is too short to enable perennial bryophytes to colonise and assume dominance, while ephemeral species thrive before the water level rises again in autumn (Furness and Hall 1981, Hugonnot 2005, Bijlsma et al. 2012). These species are considered relatively rare and threatened in Europe, for example, *Physcomitriumeurystomum* and *Ph.sphaericum* are vulnerable (VU) on the European level (Hodgetts et al. 2019), while their habitats are protected as NATURA 2000 habitats.

Besides the primary intention of the WFD to ensure water quality assessment on the national level, the implementation of monitoring in Croatia yielded a significant amount of new national bryophyte records. Through five years of intensive field surveys of Croatian freshwaters, as many as eight bryophyte species were found as new for national bryoflora: *Fissidensfontanus* (Alegro et al. 2019, Šegota et al. 2019), *Dichodontiumflavescens*, *Ricciocarposnatans* (Alegro et al. 2019), *Physcomitriumeurystomum* (Rimac et al. 2019b), *Physcomitriumsphaericum* (*Ellis et al. 2020*), *Ricciarhenana* (Ellis et al. 2020) and *Bryumklinggraeffii* and *Philonotismarchica* (Rimac et al. 2021). In addition, several rare or doubtful species with only old historical data have been confirmed (*Fissidensarnoldii*, *Hygroamblystegiumfluviatile*, *Leptobryumpyriforme*, *Physcomitriumpatens*, *Ricciacavernosa*, *R.frostii*, *R.glauca*) within this study.

The added value of our study is that, along with watercourses, we examined standing water bodies for their bryoflora as well. Altogether, nine natural lakes and 36 artificial or heavily-modified standing water bodies were studied. Bryophytes were found at five lakes and 12 artificial or heavily-modified standing water bodies. Most of the 24 recorded bryophyte species occupied shallow waters, lacustrine drawdown zones and moist riparian habitats. However, in our study, scattered populations of the rare species in the Croatian flora, *Fissidensfontanus*, were found at a depth of 2.5 m in the riverine mesotrophic Lake Visovac (the Krka River, the Dinaric–Mediterranean Subecoregion) and large colonies of *Drepanocladusaduncus* at 4 to 6 m deep water in the mesotrophic Ponikve Reservoir (the Island of Krk, the Dinaric–Mediterranean Subecoregion). Although the majority of bryophyte species cannot inhabit deep waters and they maintain terrestrial reproduction features (Vitt et al. 1986), mosses can be found within the macrophyte vegetation of lakes, even at the lower depth limit, sometimes mixed with charophytes or vascular plants (Chambers and Kalff 1985, Riis and Sand-Jensen 2017). In temperate regions, mosses were found to be particularly abundant in oligotrophic lakes (Raven 1988, Arts 1990, Srivastava et al. 1995), primarily because of the sufficient amount of light penetrating to the deeper zones of clear lakes. Although we found several truly aquatic bryophytes in our lakes and although the majority of the surveyed lakes in the Dinaric Ecoregion were oligotrophic, bryophytes were not dominant in any of the lakes surveyed. On the contrary, oligotrophic lakes were often inhabited by charophytes, which flourished in karstic lakes with basic and alkaline water (Mihaljević et al. 2020).

## Conclusions

Bryophytes are an important part of freshwater biodiversity in Croatia, inhabiting a wide variety of ecological niches associated with running and standing waters. The diversity of aquatic and semi-aquatic species is governed by the heterogeneity of different environmental factors, which determine their presence or absence, as well as the community structure. Our research revealed a quite high bryophyte diversity in aquatic and semi-aquatic habitats, with substantial differences between particular regions, especially in species richness and composition, as well as in life-form and life-strategy spectra. The Water Framework Directive not only improved the assessment of the ecological status of water bodies in Croatia by including the bryophytes as a part of macrophyte vegetation, but it has proven to be a good tool for the detection of rare, neglected or overlooked bryophyte species. This is especially important in regions where the bryophytes are still generally little researched, as in the case of southeast Europe. This study is, therefore, a valuable contribution to the knowledge of freshwater bryophyte diversity of Croatia, as well as of southeast Europe.

## Supplementary Material

E901B9E1-9A12-5932-AAFF-5B2C308A8DEA 10.3897/BDJ.10.e83902.suppl1 Supplementary material 1 Complete list of the freshwater bryoflora of Croatia with distribution data, chorological and life-trait information on the species and altitude and climatological data of the sites.
Data type Data table Brief description Coordinates are given in WGS84 system. Abbreviations: PAN–Pannonian Ecoregion, DIN CON–Dinaric Ecoregion, Continental Subecoregion, DIN MED–Dinaric Ecoregion, Mediterranean Subecoregion; life-forms: At–Aquatic trailing, De–dendroid, Le –lemnoid, Mr–rough mat, Ms–smooth mat, Mt–thalloid mat, St–solitary thalloid, Tf–turf, Ts–scattered turf, Tuft–tuft and We–weft); life strategy: p–perennials, c–colonists, pc–competitive perennials, l–long-lived shuttle, a–annual shuttle, cp–pioneer colonists, Braun-Blanquet cover and abundance classes: r = one individual, + = up to 5 individuals, 1 = up to 50 individuals, 2m = over 50 individuals, 2a = coverage 5–15%, 2b = coverage 15–25%, 3 = 25–50%; 4 = coverage 50–75%; 5 = coverage over 75%. File: oo_680458.xlsx https://binary.pensoft.net/file/680458 Rimac, A; Šegota, V; Alegro, A; Vuković, N; Koletić, N.

## Figures and Tables

**Table 1.** List of bryophyte species, along with the number of occurrences in Croatia and sub- and ecoregions. P–Pannonian Ecoregion, D–Dinaric Ecoregion, C–Continental Subecoregion, M–Mediterranean Subecoregion.

**Taxon**	**Number of sampling sites per ecoregion/subecoregion**	**Total number of sampling sites**
**Marchantiophyta**		
Jungermanniopsida		
Jungermanniales		
Jungermanniaceae		
1. *Jungermanniaatrovirens* Dumort	D: C (6), M (3)	9
Lophocoleaceae		
2. *Chiloscyphuspallescens* (Ehrh.) Dumort.	P (3); D: C (2), M (1)	6
3. *Chiloscyphuspolyanthos* (L.) Corda	P (4); D: C (12), M (7)	23
4. *Lophocoleabidentata* (L.) Dumort.	D: C (2)	2
Pelliales		
Pelliaceae		
5. *Apopelliaendiviifolia (Dicks.)* Nebel & D.Quandt	P (3); D: C (22), M (18)	43
6. *Pellianeesiana (Gottsche)* Limpr.	P (7)	7
Marchantiopsida		
Lunulariales		
Lunulariaceae		
7. *Lunulariacruciata* (L.) Dumort. ex Lindb.	P (1); D: C (1)	2
Marchantiales		
Conocephalaceae		
8. *Conocephalumsalebrosum* Szweyk., Buczk. & Odrzyk.	P (8); D: C (6), M (1)	15
Marchantiaceae		
9. *Marchantiapolymorpha* L.	P (6); D: C (16), M (2)	24
Ricciaceae		
10. *Ricciacavernosa* Hoffm.	P (4); D: C (1)	5
11. *Ricciafluitans* L.	P (6); D: C (3), M (1)	10
12. *Ricciafrostii* Austin	P (1)	1
13. *Ricciaglauca* L.	P (1)	1
14. *Ricciarhenana* Lorb. ex Müll.Frib.	P (3)	3
15. *Ricciocarposnatans* (L.) Corda	P (2)	2
**Bryophyta**		
Bryopsida		
Bartramiales		
Bartramiaceae		
16. *Philonotismarchica* (Hedw.) Brid.	D: C (1)	1
Bryales		
Bryaceae		
17. *Bryumargenteum* Hedw.	P (4); D: C (1)	5
18. *Bryumbarnesii* J.B. Wood ex Schimp.	D: M (1)	1
19. *Bryumdichotomum* Hedw.	P (1); D: M (1)	2
20. *Bryumklinggraeffii* Schimp.	P (2)	2
21. *Bryumruderale* Crundw. & Nyholm	D: C (1)	1
22. *Ptychostomumpseudotriquetrum* (Hedw.) J.R.Spence & H.P.Ramsay ex Holyoak & N.Pedersen	P (8); D: C (10), M (7)	25
Mniaceae		
23. *Mniummarginatum* (Dicks.) P.Beauv.	D: C (3)	3
24. *Plagiomniumaffine* (Blandow ex Funck) T.J.Kop.	D: C (1)	1
25. *Plagiomniumelatum* (Bruch et Schimp.) T.J.Kop.	P (1)	1
26. *Plagiomniumellipticum* (Brid.) T.J.Kop.	P (1); D: C (1)	2
27. *Plagiomniumundulatum* (Hedw.) T.J.Kop.	P (3); D: C (7)	10
28. *Pohliamelanodon* (Brid.) A.J.Shaw	P (12); D: C (2), M (2)	16
29. *Rhizomniumpunctatum* (Hedw.) T.J.Kop.	P (1); D: C (1)	2
Dicranales		
Amphidiaceae		
30. *Dichodontiumflavescens* (Dicks.) Lindb.	P (1); D: C (4)	5
31. *Dichodontiumpellucidum* (Hedw.) Schimp.	P (2); D: C (3)	5
32. *Dicranellavaria* (Hedw.) Schimp.	P (5); D: C (1), M (2)	8
Fissidentaceae		
33. *Fissidensadianthoides* Hedw.	P (4); D: C (1)	5
34. *Fissidensarnoldii* R.Ruthe	D: C (1)	1
35. *Fissidenscrassipes* Wilson ex Bruch & Schimp.	P (6); D: C (19), M (14)	39
36. *Fissidensfontanus* (Bach.Pyl.) Steud.	P (3); D: M (1)	4
37. *Fissidensgracilifolius* Brugg.-Nann. & Nyholm	D: C (1), M (2)	3
38. *Fissidenspusillus* (Wilson) Milde	P (5)	5
39. *Fissidenstaxifolius* Hedw.	P (2); D: C (1), M (2)	5
Pottiaceae		
40. *Barbulaunguiculata* Hedw.	P (1); D: C (2)	3
41. *Bryoerythrophyllumrecurvirostrum* (Hedw.) P.C.Chen	D: C (1)	1
42. *Cinclidotusaquaticus* (Hedw.) Bruch & Schimp.	D: C (20), M (16)	36
43. *Cinclidotusfontinaloides* (Hedw.) P.Beauv.	P (2); D: C (28), M (21)	51
44. *Cinclidotusriparius* (Host ex Brid.) Arn.	P (7); D: C (22), M (13)	42
45. *Didymodonfallax* (Hedw.) R.H.Zander	D: C (6), M (1)	7
46. *Didymodoninsulanus* (De Not.) M.O.Hill	D: C (1)	1
47. *Didymodonluridus* Hornsch.	D: C (1), M (2)	3
48. *Didymodonspadiceus* (Mitt.) Limpr.	D: C (3)	3
49. *Didymodontophaceus* (Brid.) Lisa	D: C (2), M (8)	10
50. *Eucladiumverticillatum* (With.) Bruch & Schimp.	D: C (6), M (4)	10
51. *Gymnostomumaeruginosum* Sm.	D: C (1)	1
52. *Hymenostyliumrecurvirostrum* (Hedw.) Dixon	D: C (3)	3
53. *Trichostomumcrispulum* Bruch	D: C (1)	1
Funariales		
Funariaceae		
54. *Funariahygrometrica* Hedw.	P (1); D: C (3), M (2)	6
55. *Physcomitriumpatens* (Hedw.) Mitt.	P (7); D: C (1)	8
56. *Physcomitriumeurystomum* Sendtn.	P (1)	1
57. *Physcomitriumsphaericum* (C.F.Ludw. ex Schkur.) Brid.	P (1)	1
Hypnales		
Amblystegiaceae		
58. *Cratoneuronfilicinum* (Hedw.) Spruce	P (8); D: C (34), M (25)	67
59. *Drepanocladusaduncus* (Hedw.) Warnst.	P (2); D: C (2), M (4)	8
60. *Hygroamblystegiumfluviatile* (Hedw.) Loeske	P (1)	1
61. *Hygroamblystegiumhumile* (P.Beauv.) Vanderp., Goffinet & Hedenäs	P (1)	1
62. *Hygroamblystegiumtenax* (Hedw.) Jenn.	P (2); D: C (1), M (1)	4
63. *Hygroamblystegiumvarium* (Hedw.) Mönk.	P (2); D: C (3)	5
64. *Hygrohypnumluridum* (Hedw.) Jenn.	D: C (4)	4
65. *Leptodictyumriparium* (Hedw.) Warnst.	P (42); D: C (19), M (8)	69
66. *Palustriellacommutata* (Hedw.) Ochyra	P (2); D: C (3), M (4)	9
67. *Palustriellafalcata* (Brid.) Hedenäs	P (1); D: C (6), M (3)	10
Brachytheciaceae		
68. *Brachytheciummildeanum* (Schimp.) Schimp.	P (3); D: C (2)	5
69. *Brachytheciumrivulare* Schimp.	P (2); D: C (10), M (3)	15
70. *Brachytheciumrutabulum* (Hedw.) Schimp.	P (4); D: C (6), M (1)	11
71. *Brachytheciumsalebrosum* (Hoffm. ex F.Weber et D.Mohr) Schimp.	D: C (1), M (1)	1
72. *Oxyrrhynchiumhians* (Hedw.) Loeske	P (8); D: C (4)	12
73. *Oxyrrhynchiumschleicheri* (R.Hedw.) Röll	D: M (1)	1
74. *Oxyrrhynchiumspeciosum* (Brid.) Warnst.	P (6); D: C (4), M (1)	11
75. *Rhynchostegiellacurviseta* (Brid.) Limpr.	D: C (1)	1
76. *Rhynchostegiellateneriffae* (Mont.) Dirkse & Bouman	D: C (3)	3
77. *Rhynchostegiumriparioides* (Hedw.) Cardot	P (19); D: C (38), M (36)	93
Fontinalaceae		
78. *Fontinalisantipyretica* Hedw.	P (20); D: C (43), M (47)	110
79. Fontinalishypnoidesvar.duriaei (Schimp.) Kindb.	P (1); D: C (2)	3
Leskeaceae		
80. *Leskeapolycarpa* Hedw.	P (1); D: C (1), M (2)	4
Neckeraceae		
81. *Thamnobryumalopecurum* (Hedw.) Gangulee	D: C (3), M (2)	5
Pylaisiaceae		
82. *Calliergonellacuspidata* (Hedw.) Loeske	P (1); D: C (7), M (1)	9
Splachnales		
Meesiaceae		
83. *Leptobryumpyriforme* (Hedw.) Wilson	P (2)	2

**Table 2.** Comparison amongst Croatian ecoregions and subecoregions.

	**(sub)ecoregion**			
	**Pannonian**	**Dinaric**	**Dinaric–Continental**	**Dinaric**– **Mediterranean**
**Total number of bryophyte** **species**	**57**	**70**	**65**	**40**
**Mosses (Bryophyta)**	44	60	55	33
pleurocarpous	19	23	21	16
acrocarpous	25	37	34	17
**Liverworts**	15	15	10	7
leafy	4	4	4	3
thallose	11	11	6	4
dominant species*	*Lept rip*	*Fon ant, Rhy rip, Cra fil, Cin fon*	*Fon ant, Rhy rip, Cra fil, Cin fon, Cin rip, Apo end*	*Fon ant, Rhy rip*, *Cra fil*
coverage % (mean)	2.40	3.60	4.04	4.15
species richness (total, mean ± SE)	3.40 ± 0.35	4.60 ± 0.33	5.90 ± 0.58	3.40 ± 0.29
range (min–max)	1–15	1–20	1–20	1–12
**Families**				
number of families	18	20	20	19
dominant families**	*Amb, Bra, Ricc, Fiss, Mni*.	*Pott, Brac, Ambl, Fiss, Mnia*	*Pott, Brac, Ambl, Mnia, Fiss*,	*Pott, Ambl, Brac*, *Fiss, Brya*
**Watercourses/sampling sites**	**68/76**	**85/132**	**43/62**	**42/70**
rivers/sampling sites	50/56	69/107	39/57	30/50
artificial and heavily-modified watercourses / sampling sites	18/20	16/25	4/5	12/20
**Standing waters/sampling sites**	**3/3**	**14/17**	**6/8**	**8/9**
natural lakes/ sampling sites	-	5/5	3/3	2/2
artificial or heavily-modified standing waters / sampling sites	3/3	9/12	3/5	6/7
**Altitude (m a.s.l.)**				
mean ± SE	146 ± 8.23	231 ± 13.53	310 ± 18.32	162 ± 16.15
range (min-max)	81–547	1–711	111–703	1–711
**Climate**				
mean annual air temperature (°C) (± SE)	11.6 ± 0.1	12.4 ± 0.1	10.9 ± 0.1	13.7 ± 0.1
mean daily mean air temperatures of the wettest quarter (°C) (± SE)	17.4 ± 0.4	11.1 ± 0.2	11.6 ± 0.3	10.7 ± 0.3
mean daily mean air temperatures of the driest quarter (°C) (± SE)	3.3 ± 0.1	14.1 ± 0.8	6.5 ± 0.9	20.9 ± 0.6
annual precipitation amount (kg/m^2^) (mean ± SE)	935.3 ± 14.6	1360.2 ± 20.5	1415.1 ± 24.7	1311.5 ± 31.0
